# Cytosine Deaminase-TRAIL Expressing Human Adipose Stem Cells Inhibit Tumor Growth in Castration Resistant Prostate Cancer Bearing Mice with Less Toxicity

**DOI:** 10.3390/ijms27031563

**Published:** 2026-02-05

**Authors:** Jae Heon Kim, Hyun Young Lee, In Seok Hong, Jeongkun Lee, Sang Hun Lee, Yun Seob Song

**Affiliations:** 1 Department of Urology, Soonchunhyang University Seoul Hospital, Soonchunhyang University Medical College, Seoul 04401, Republic of Korea; piacekjh@hanmail.net (J.H.K.); amoke@naver.com (H.Y.L.); 2 Department of Chemistry, Kongju National University, Kongju 32588, Republic of Korea; ishong@kongju.ac.kr; 3 Program in Biomedical Sciences, Engineering, Department of Biomedical Sciences, College of Medicine, Inha University, Incheon 22332, Republic of Korea; jeongkun@inha.ac.kr

**Keywords:** adipose stem cells, castration-resistant prostate cancer, gene-directed enzyme prodrug therapy, cytosine deaminase, TRAIL, tumor tropism, hTERT-immortalization, suicide gene

## Abstract

Stem cells can selectively migrate toward cancer cells, and therapeutic genes can be introduced into stem cells. Tumor necrosis factor-related apoptosis-inducing ligand (TRAIL) induces apoptosis in cancer cells without harming normal cells. In this study, we evaluated the inhibition of tumor growth in castration-resistant prostate cancer (CRPC) using human adipose-derived stem cells (ADSCs) engineered to express cytosine deaminase (CD) and soluble TRAIL (sTRAIL), combined with the prodrug 5-fluorocytosine (5-FC). An immortalized human ADSC line (hTERT-ADSC) was transduced with a lentiviral vector encoding CD and sTRAIL, generating ADSC.CD.sTRAIL cells. Expression of chemoattractant ligands and receptors was assessed by RT-PCR. The suicide gene effect was evaluated by 5-FC treatment, measuring cell viability and apoptosis markers in vitro. A subcutaneous CRPC mouse model was used for in vivo studies. ADSC.CD.sTRAIL cells showed enhanced migration toward prostate cancer cells. Treatment with 5-FC significantly reduced cell viability, and co-culture with PC3 cells plus 5-FC increased apoptosis marker expression. In vivo, mice treated with ADSC.CD.sTRAIL and 5-FC had significantly smaller tumor volumes than control groups, with no treatment-related toxicity observed. These findings suggest that ADSCs overexpressing CD and sTRAIL, combined with 5-FC, effectively inhibit CRPC tumor growth and represent a promising targeted therapeutic strategy.

## 1. Introduction

Advanced prostate cancer is initially responsive to androgen deprivation therapy; however, within 12–18 months, most tumors progress to castration-resistant prostate cancer (CRPC). Once CRPC develops, the prognosis is poor, with a median survival of approximately two years and only palliative options available [1]. Conventional chemotherapy has limited efficacy in this setting, as dose intensification is restricted by severe systemic toxicity. To overcome these challenges, gene-directed enzyme prodrug therapy (GDEPT) has emerged as an attractive strategy. In this approach, a prodrug-activating enzyme is selectively delivered to tumor cells, where it converts a nontoxic prodrug into a cytotoxic drug, achieving localized therapeutic effects while reducing systemic toxicity. One of the most studied enzymes in GDEPT is cytosine deaminase (CD), a bacterial enzyme that converts 5-fluorocytosine (5-FC) into the chemotherapeutic 5-fluorouracil (5-FU) [2,3]. This system allows high local concentrations of 5-FU in the tumor microenvironment while minimizing systemic exposure.

Efficient gene delivery to tumors remains a key challenge for cancer gene therapy. Mesenchymal stem cells (MSCs), including adipose-derived stem cells (ADSCs), exhibit natural tumor-tropic properties and are promising vehicles for delivering suicide genes [4,5,6,7,8,9]. Previous studies have demonstrated that stem cell-mediated CD/5-FC therapy can inhibit tumor growth [10,11]. For example, prostate cancer cells engineered with a CD–uracil phosphoribosyltransferase (UPRT) fusion gene showed markedly reduced growth following 5-FC treatment [12,13,14]. Similarly, we have shown that human neural stem cells expressing CD (HB1.F3.CD) migrated to prostate tumors and, when combined with systemic 5-FC, significantly suppressed tumor volumes in mice [15]. However, the use of neural stem cells faces practical and ethical barriers, including challenges in procurement, reproducibility, and clinical translation.

ADSCs represent an advantageous alternative for clinical applications. They can be isolated from patients with minimal invasiveness, expanded ex vivo to therapeutic numbers, genetically modified efficiently, and are free from ethical concerns [16]. Building on these advantages, a tumor-targeted gene therapy platform was developed using ADSCs as delivery vehicles [17]. In this strategy, human telomerase reverse transcriptase (hTERT)-immortalized ADSCs were engineered to stably express both CD and soluble TRAIL (sTRAIL), creating ADSC.CD.sTRAIL cells. TRAIL is a cytokine that selectively induces apoptosis in malignant cells with minimal toxicity to normal tissues [18,19]. Modified soluble trimeric TRAIL proteins, designed with a secretion signal, a trimerization domain, and the receptor-binding region, have been shown to enhance apoptotic activity compared with conventional TRAIL proteins [20]. Importantly, combining TRAIL with 5-FU has demonstrated synergistic antitumor effects in several malignancies, including prostate cancer [21,22]. This suggests that a combined CD/5-FC plus TRAIL approach may maximize tumor cell death without exacerbating systemic toxicity.

Building on our prior stem cell-based CD/5-FC approach [11,13], here we introduce TRAIL into the same platform to engage the extrinsic (death receptor-mediated) pathway of apoptosis in addition to the intrinsic pathway triggered by 5-FU. We anticipated that incorporating TRAIL would provide direct pro-apoptotic activity against cancer cells (via caspase-8 activation) alongside the bystander killing by 5-FU, leading to more effective tumor cell death without added systemic toxicity. We hypothesized that ADSC.CD.sTRAIL cells would migrate to prostate tumors, convert 5-FC to 5-FU in the tumor microenvironment, continuously secrete TRAIL, and thereby induce apoptosis in tumor cells while minimizing side effects.

This study is a proof-of-concept investigation to evaluate whether hTERT-immortalized ADSCs overexpressing CD and sTRAIL, combined with 5-FC treatment, can inhibit the growth of CRPC in a mouse model while maintaining safety.

## 2. Results

### 2.1. Generation and Characterization of hTERT-ADSC.CD.sTRAIL Cells

**Figure 1:**
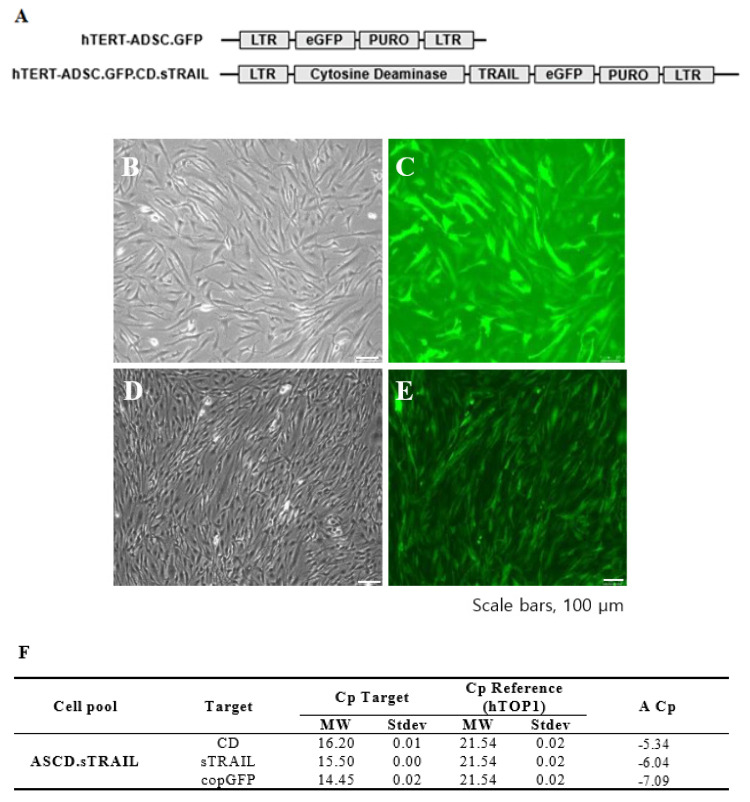
CD.sTRAIL overexpressing hTERT-immortalized human adipose stem cell line (hTERT-hADSC.CD.sTRAIL). (**A**): hTERT-hADSC and hTERT-hADSC.CD.sTRAIL were generated via lentiviral transduction of GFP, CD, sTRAIL gene using CLV-Ubic vector. (**B**): Phase contrast microscopy of hTERT-hADSC.GFP cells. (**C**): Immunofluorescence microscopy of hTERT-hADSC.GFP cells. (**D**): Phase contrast microscopy of hTERT-hADSC.GFP.CD.sTRAIL cells. (**E**): Immunofluorescence microscopy of hTERT-hADSC.GFP.CD.sTRAIL cells. (**F**): Real-time PCR. ΔC_p_ of CD and sTRAIL was −5.34 and −6.04. ADCS = hTERT-hADSC.

We successfully established an hTERT-immortalized human ADSC line (ASC52-Telo) and generated ADSCs expressing CD and sTRAIL via lentiviral transduction. The parental hTERT-ADSC and the engineered ADSC.CD.sTRAIL line are shown in Figure 1. Phase contrast microscopy revealed that both cell lines displayed the typical spindle-shaped morphology of MSCs (Figure 1B for hTERT-ADSC; Figure 1C for hTERT-ADSC.CD.sTRAIL), with no obvious morphological differences after immortalization or gene introduction. The ADSC.CD.sTRAIL cells expressed GFP (encoded by the vector as a reporter) efficiently, as seen by fluorescence microscopy (Figure 1D,E), indicating a high transduction rate.

**Figure 2:**
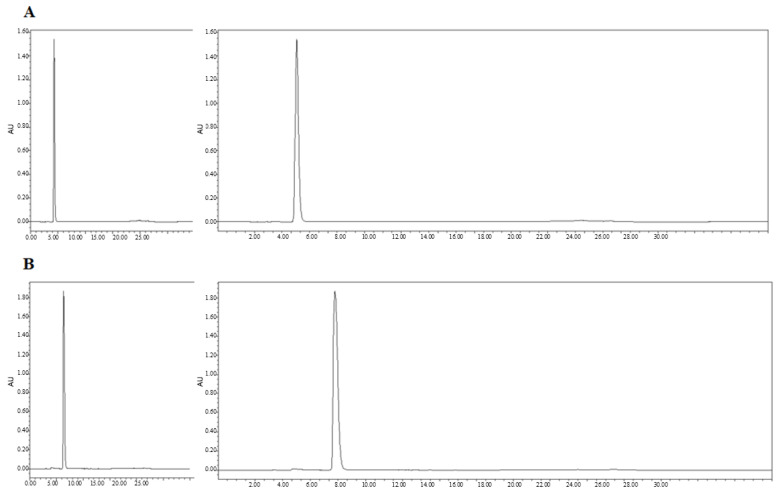

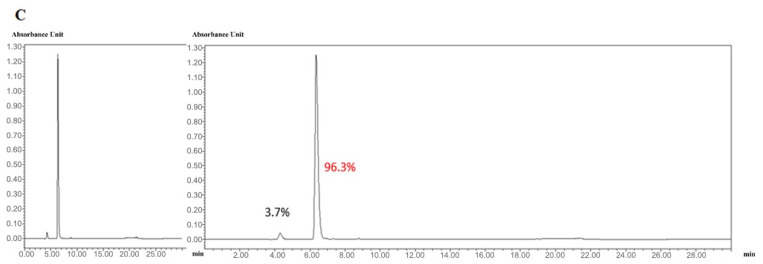
High performance liquid chromatography (HPLC). HPLC analysis showed that 5-FC (**A**) and 5-FU (**B**) eluted at 4.3 and 6.3 min, respectively. The percentages of 5-FC and 5-FU were 3.7 and 96.3 (**C**). Flow = 1.0, Water(A)% = 100, MeCN(B)% = 0. *X*-axis: min. *Y*-axis: Absorbance Unit.

We next confirmed the expression of the CD and sTRAIL transgenes in ADSC.CD.sTRAIL cells. Quantitative real-time PCR (qRT-PCR) showed robust overexpression of both CD and sTRAIL mRNA in ADSC.CD.sTRAIL compared to parental cells (which have no CD or TRAIL transgene). The threshold cycle difference (ΔC_p_) was −5.32 for CD and −6.04 for sTRAIL (Figure 1F), indicating approximately 40–64-fold higher transcript levels in ADSC.CD.sTRAIL cells relative to parental hTERT-ADSCs. Western blot analysis for the CD protein was attempted; however, due to the lack of a reliable antibody against the E. coli CD enzyme, we could not detect a specific CD band in cell lysates (as expected). Instead, we demonstrated functional CD enzyme activity by an HPLC-based conversion assay: in conditioned medium from ADSC.CD.sTRAIL cultures, 96.3% of 5-FC was converted to 5-FU within 24 h, whereas no conversion occurred in medium from parental ADSCs (Figure 2A,B). This functional assay confirms that the CD gene is not only present but actively expressed at the protein level (producing enzyme activity). For sTRAIL, we did not perform ELISA or immunofluorescence staining of ADSC.CD.sTRAIL cells to detect human TRAIL protein. However, we confirmed transgene introduction in engineered ADSCs by PCR. After RT-PCR using sTRAIL-specific primers and agarose gel electrophoresis, an sTRAIL amplicon was detected only in ADSC.sTRAIL and not in ADSC. Taken together, these results confirmed that our engineered ADSCs express the CD/5-FC → 5-FU “suicide” gene and the sTRAIL gene robustly at the mRNA level and produce functional protein outputs (5-FU generation and secreted TRAIL, respectively).

### 2.2. Immunophenotype and Migration Properties of ADSC.CD.sTRAIL

**Figure 3:**
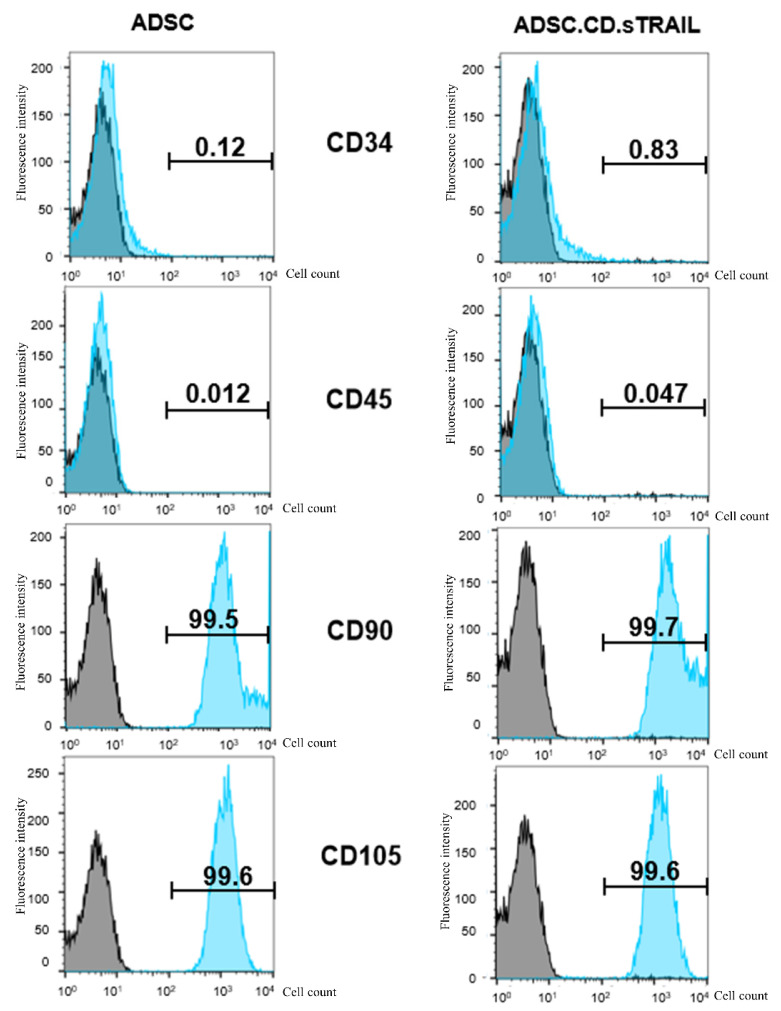
Flow cytometric characterization of parental hTERT-ADSC and ADSC.CD.sTRAIL cells. Representative flow histograms for surface markers. Blue peaks = specific antibody staining; gray peaks = isotype control. Both cell types were strongly positive for MSC markers CD90 and CD105, and negative for hematopoietic markers CD34 and CD45. These results confirm maintenance of the MSC phenotype after immortalization and genetic modification. Abbreviations: ADSC—adipose-derived stem cell.

It was critical to verify that hTERT immortalization and genetic modification did not alter the MSC identity of the ADSCs or endow them with unwanted traits. Flow cytometric characterization demonstrated that both the parental hTERT-ADSC and the ADSC.CD.sTRAIL cells retained a typical MSC immunophenotype (Figure 3). Specifically, the cells were positive for MSC-associated surface markers CD90 and CD105 (blue histograms distinctly shifted relative to isotype controls in gray), and negative for hematopoietic markers CD34 and CD45 (blue histograms overlapped with gray). The expression levels of CD90 and CD105 were unchanged after immortalization and transduction, indicating preserved phenotype. These data suggest that the cells did not undergo spontaneous dedifferentiation or transformation; they maintained MSC characteristics post-modification. Notably, the ADSC.CD.sTRAIL cells showed stable growth with no abnormal morphologies through prolonged culture.

**Figure 4:**
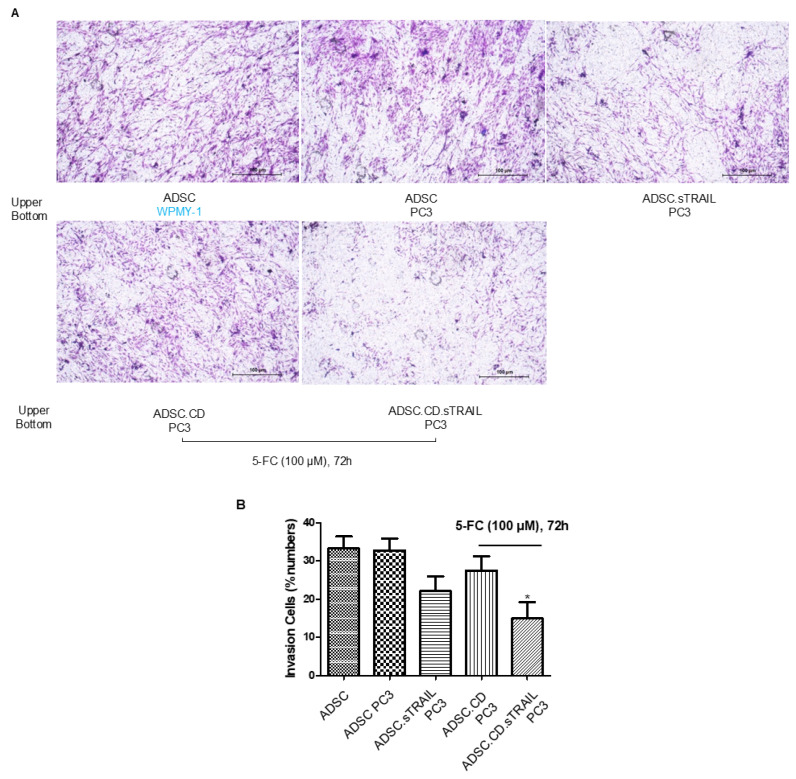
hTERT-ADSC.CD.sTRAIL line exhibits tumor-tropic migration toward prostate cancer. (**A**) Transwell invasion assay: ADSCs were placed in the upper chamber with Matrigel, and PC3 prostate cancer cells (or control WPMY-1 stromal cells) were in the lower chamber as a chemoattractant source. After 48 h, migrated ADSCs were stained and counted. Representative images show more cells migrating toward PC3-conditioned medium. (**B**) Quantification of migration: Both parental hTERT-ADSC and ADSC.CD.sTRAIL showed significantly higher migration toward PC3 (*p* < 0.05). There was no significant difference between parental and CD.sTRAIL-modified ADSCs in migratory index although a slight decrease with 5-FC exposure was noted. ADSC.sTRAIL cells exhibited also strong migratory tropism toward PC3 prostate cancer cell-conditioned medium (*p* < 0.05). Quantitatively, ADSC.CD.sTRAIL with 5-FC toward PC3 showed reduced migration than ADSC.sTRAIL (*p* < 0.05), However, their migration toward PC3 was higher than toward control medium (*p* < 0.05). Data are mean ± SE, *n* = 3 independent experiments. Abbreviations: PC3—prostate cancer cell line; WPMY-1—prostate stromal cell line (control); 5-FC—5-fluorocytosine. * *p* < 0.05, ADSC.CD.sTRAIL with 5-FC vs. control WPMY-1 stromal cells.

We next assessed the tumor-tropic migration ability of the ADSC.CD.sTRAIL cells, as effective homing is pivotal for delivering therapeutic genes to tumors. In transwell invasion assays (Figure 4A), both parental hTERT-ADSCs and ADSC.CD.sTRAIL cells exhibited strong migratory tropism toward PC3 prostate cancer cell-conditioned medium. ADSC.sTRAIL cells exhibited also strong migratory tropism toward PC3 prostate cancer cell-conditioned medium. Quantitatively, ADSC.CD.sTRAIL with 5-FC toward PC3 showed reduced migration compared to ADSC.sTRAIL; their migration toward PC3 was ~4.2-fold higher than toward control medium (fibroblast WPMY-1 conditioned medium), which was comparable to the parental ADSC’s enhancement (~4.8-fold)—indicating that introducing CD/TRAIL did not impair the cells’ migratory responsiveness. In fact, ADSC.CD.sTRAIL cells showed slightly higher migration than parental ADSCs (Figure 4B), although migration was somewhat reduced if 5-FU was present (likely because some ADSC.CD.sTRAIL undergo self-killing when converting 5-FC to 5-FU; see below). These results demonstrate that the engineered ADSCs maintain the inherent tumor-seeking behavior of MSCs.

**Figure 5:**
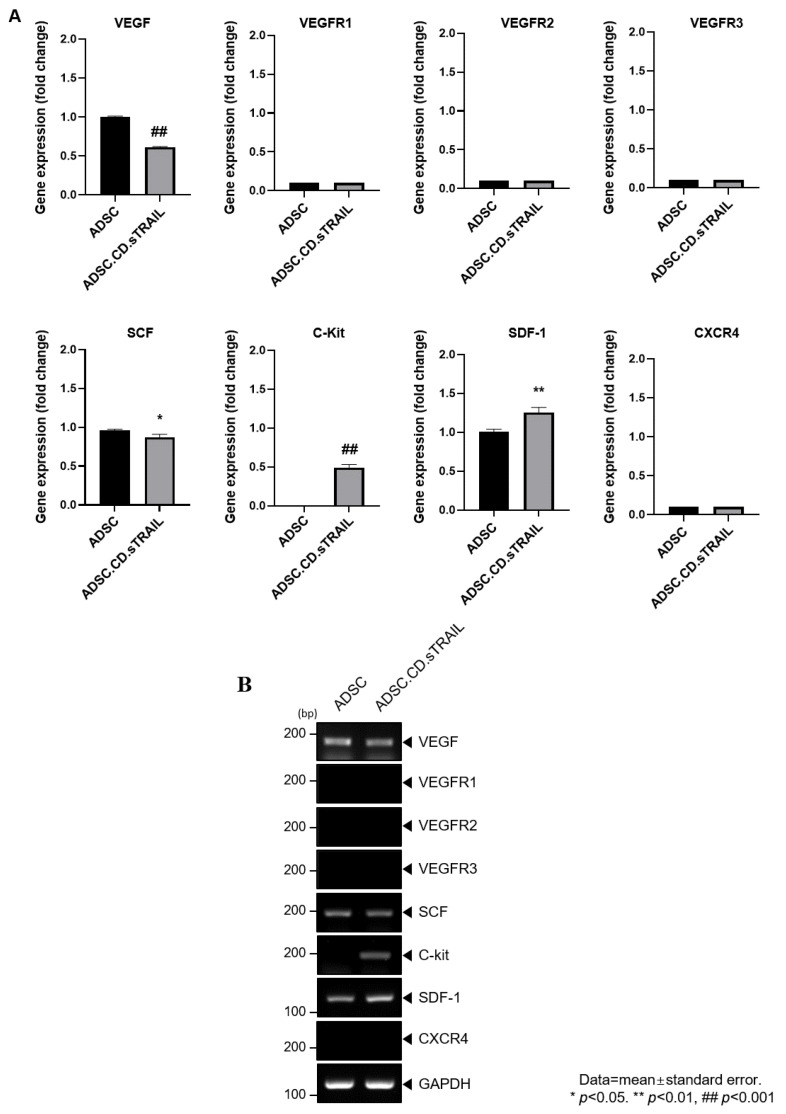
Graphical representation of migrant hTERT-ADSC.CD.sTRAIL line. Quantitative expression of chemoattractant factors. (**A**): Using RT-PCR, the presence of ligands, VEGF, SCF, SDF-1and their corresponding receptors c-kit in ADSC.CD.sTRAIL cells, was confirmed. (**B**): RT-PCR of chemoattractant factors. Using real-time PCR, the level of c-kit, SDF-1 increased. ADCS = hTERT-hADSC, stem cell factor = SCF, stromal cell-derived factor = SDF, vascular endothelial growth factor = VEGF, vascular endothelial growth factor receptor = VEGFR. Data = mean ± standard error.

To explore molecular factors underlying the MSC tropism, we analyzed the expression of chemoattractant ligands and their receptors in ADSC.CD.sTRAIL cells versus parental ADSCs. RT-PCR screening (Figure 5A) revealed that ADSC.CD.sTRAIL cells express various relevant factors: vascular endothelial growth factor (VEGF) and its receptors (VEGFR1, VEGFR2, VEGFR3), stem cell factor (SCF) and its receptor c-Kit, and stromal cell-derived factor 1 (SDF-1/CXCL12) and its receptor CXCR4. Notably, quantitative real-time PCR showed significantly higher mRNA levels of c-Kit and SDF-1 in ADSC.CD.sTRAIL cells compared to parental ADSCs (*p* < 0.05, Figure 5B). For example, SDF-1 (a chemokine that can attract CXCR4-expressing tumor cells or vice versa) was ~2-fold upregulated. This upregulation could enhance bidirectional signaling interactions with tumors. The increased c-Kit expression on ADSC.CD.sTRAIL is also interesting, since its ligand SCF is often secreted by tumors or injured tissue and may promote migration. Taken together, these data suggest that hTERT-immortalized, CD/TRAIL-expressing ADSCs preserve and potentially augment their tumor-homing molecular repertoire, which may partly explain their efficient migration toward prostate cancer cells in vitro.

### 2.3. In Vitro 5-FC Cytotoxicity and Induced Apoptosis

**Figure 6:**
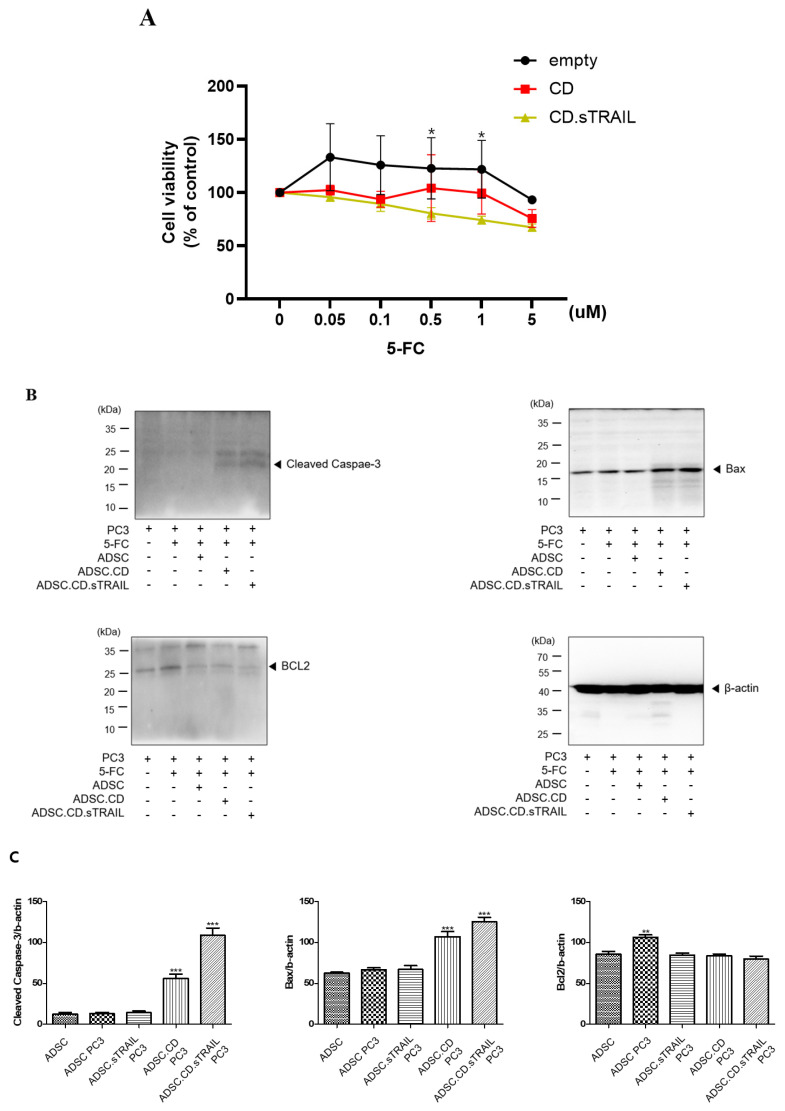
In vitro effect of hTERT-ADSC.CD.sTRAIL under the treatment of 5-FC. (**A**): The suicide effect. At more than 0.05 μM 5-FC, cell viability of ADSC.CD.sTRAIL was lower than that of ADSC. * *p* < 0.05. (**B**): apoptotic PC3 cells increased in the presence of ADSC.CD.sTRAIL under 5-FC compared with 5-FC monotherapy. (**C**): Quantitative expression of apoptotic PC3 cells. ADCS = hTERT-hADSC, ADSC.CE = CE overexpressing hTERT-immortalized human adipose stem cells. ** *p* < 0.01, *** *p* < 0.001.

We evaluated whether the CD/5-FC “suicide” system functioned as intended in our ADSC.CD.sTRAIL cells and whether the addition of TRAIL provided any synergistic benefit in killing cancer cells. First, we tested the effect of 5-FC on the ADSC.CD.sTRAIL cells themselves (suicide effect) via an MTT assay. ADSC.CD.sTRAIL viability decreased in a 5-FC dose-dependent manner (0–5 µM range), whereas parental hTERT-ADSC viability was unaffected by 5-FC (since they cannot convert it to 5-FU) (Figure 6A). At 5 µM 5-FC, ADSC.CD.sTRAIL cell viability dropped to ~55% of untreated, confirming that the CD gene is actively converting 5-FC to toxic 5-FU, which in turn kills the CD-expressing cells (a built-in safety to prevent unlimited cell proliferation).

Next, we co-cultured PC3 prostate cancer cells with our engineered ADSCs to examine the bystander killing effect on tumor cells. Co-cultures were set up at a 1:1 ratio of PC3:ADSC and treated with 5-FC (5 µM) for 3 days. We compared several conditions: PC3 + parental ADSC + 5-FC (essentially 5-FC alone, no conversion); PC3 + ADSC.CD + 5-FC (enzyme conversion, no TRAIL); and PC3 + ADSC.CD.sTRAIL + 5-FC (enzyme conversion + TRAIL). PC3 cells co-cultured with ADSC.CD.sTRAIL + 5-FC showed the greatest reduction in viable cell numbers, whereas those with ADSC.CD + 5-FC had intermediate reduction, and those with parental ADSC + 5-FC showed no reduction (similar to PC3 with 5-FC alone) (Figure 2C). Quantitatively, after 72 h, PC3 viability (by MTT) was ~35% of control in the ADSC.CD.sTRAIL + 5-FC group, vs. ~60% of control in the ADSC.CD + 5-FC group, vs. ~95–100% in the 5-FC alone group. Thus, having the CD enzyme alone significantly impaired PC3 growth (*p* < 0.01 vs. 5-FC alone, *n* = 4), and having CD + TRAIL together further enhanced tumor cell kill (*p* < 0.05 vs. CD alone). These results indicate a synergistic effect of the combined therapy: TRAIL’s pro-apoptotic action adds to the cytotoxic impact of 5-FU produced by CD.

To confirm that tumor cell death was occurring via apoptosis pathways, we analyzed apoptotic markers in PC3 cells from the co-cultures. Western blot of PC3 cell lysates (Figure 6B) showed that co-culture with ADSC.CD.sTRAIL + 5-FC led to a marked increase in the pro-apoptotic protein BAX (by ~2.1-fold relative to PC3 + 5-FC alone) and a decrease in the anti-apoptotic protein BCL-2 (to ~50% of the level in controls). Consequently, the BAX/BCL-2 ratio was substantially elevated in treated PC3 cells, consistent with activation of intrinsic apoptosis. We attempted to detect cleaved Caspase-3 in these lysates as a readout of executioner caspase activation. As shown in Figure 6B, a band corresponding to cleaved Caspase-3 (~17 kDa) was faintly visible in the ADSC.CD.sTRAIL + 5-FC group, but not in controls; however, a stronger band above it (~35 kDa) representing pro-Caspase-3 or a non-specific protein was present in all samples. We interpreted this with caution: while there is some evidence of Caspase-3 activation, the result was not clear-cut due to the non-specific band. We therefore rely on the BAX upregulation and BCL-2 reduction as evidence that apoptosis is induced. In summary, the in vitro findings demonstrate that: (1) ADSC.CD.sTRAIL can convert 5-FC to 5-FU and induce self-killing, serving as a safety mechanism; (2) they can exert a bystander killing effect on neighboring cancer cells via 5-FU; and (3) the presence of TRAIL augments tumor cell apoptosis beyond what 5-FU alone achieves, as reflected in greater viability loss and apoptotic protein changes.

### 2.4. In Vivo Efficacy of ADSC.CD.sTRAIL in a CRPC Mouse Model

We next tested the therapeutic efficacy of the engineered ADSCs in an in vivo model of CRPC. Male athymic nude mice bearing subcutaneous PC3 tumors were randomized into three groups (*n* = 5 per group): 1. Control (PBS + 5-FC)—mice received an intracardiac injection of PBS (vehicle for cells) and subsequent 5-FC prodrug treatments (to control for any 5-FC direct effects). 2. ADSC.CD + 5-FC—mice received 1 × 10^6^ hTERT-ADSC.CD cells (no TRAIL) via intracardiac injection, then 5-FC treatments. 3. ADSC.CD.sTRAIL + 5-FC—mice received 1 × 10^6^ hTERT-ADSC.CD.sTRAIL cells via intracardiac injection, then 5-FC.

**Figure 7:**
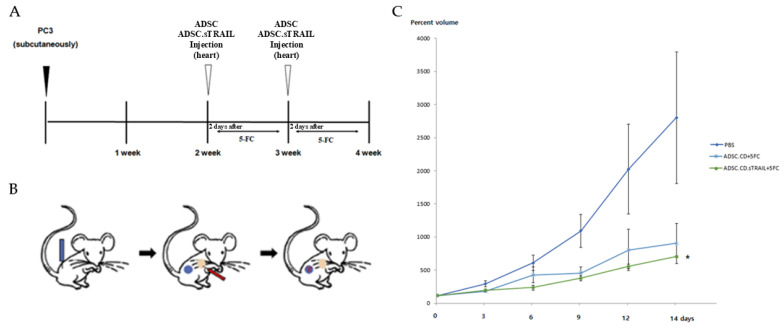
Therapeutic efficacy of ADSC.CD.sTRAIL with 5-FC in vivo (PC3 CRPC subcutaneous tumor model). Male nude mice (*n* = 5 per group) with established PC3 tumors were treated as follows: PBS + 5-FC (control), ADSC.CD + 5-FC, or ADSC.CD.sTRAIL + 5-FC. 5-FC (500 mg/kg IP) was given daily (5 days on, 2 off) for 14 days to all groups. (**A**) Schematic summary of the treatment. (**B**): Illustration of the induction of prostate cancer using PC3, systemic injection of hTERT-ADSC.CD. sTRAIL cells and migration of the gene-modified stem cells toward the prostate cancer. Blue: PC3; red: hTERT-ADSC.CD.sTRAIL cells. (**C**): The inhibitory effects of 5-FC showed better effects with ADSC.CD.sTRAIL +5-FC than those with ADSC.CD or 5-FC alone. Data are mean ± SE. Statistical notes: Two-way ANOVA showed a significant interaction of treatment and time; * *p* < 0.05. *X*-axis: date, *Y*-axis: Percent volume means tumor volume at each time point expressed as a percentage of the initial tumor volume prior to treatment. hTERT-ADSC.CD.sTRAIL = CD and TRAIL overexpressing hTERT-immortalized human adipose stem cells.

One day after cell (or PBS) injection, all mice were started on 5-FC intraperitoneal administration (500 mg/kg/day). The 5-FC was given in cycles of 5 days on, 2 days off, for two weeks total. Tumor volumes were measured at baseline (day 0) and then every 2–3 days until day 14. Figure 7 shows the tumor growth in each individual mouse, and summarizes the final tumor volumes across groups. By the end of the experiment (day 14), the ADSC.CD.sTRAIL + 5-FC treated mice had dramatically smaller tumors compared to the other groups. In relative terms, tumors in the ADSC.CD.sTRAIL group were only ~26% of the size of control tumors on day 14.

Statistical analysis confirmed a significant treatment effect (*p* < 0.01 by ANOVA). Post hoc tests showed that ADSC.CD.sTRAIL + 5-FC achieved significantly greater tumor inhibition than ADSC.CD + 5-FC (*p* < 0.05) and PBS + 5-FC (*p* < 0.01). The ADSC.CD + 5-FC group also had smaller tumors than control, though the difference was more modest (~29% reduction, not reaching significance at *p* = 0.07, likely due to the small sample). These outcomes indicate that the combination of CD/5-FC and TRAIL delivered by ADSCs was the most effective, consistent with our in vitro synergy results.

Figure 7C illustrates the tumor growth curves over time as a percentage of initial tumor volume. In the PBS + 5-FC group, tumors grew steadily by day 14. In the ADSC.CD + 5-FC group, tumor growth slowed. In contrast, the ADSC.CD.sTRAIL + 5-FC group showed the inhibition of tumor growth by day 14. This temporal pattern underscores that TRAIL provided an early and potent apoptotic stimulus, leading to tumor shrinkage rather than just growth delay.

Importantly, throughout the treatment, no signs of systemic toxicity were observed. All mice survived to the endpoint and no obvious behavioral changes were observed. The absence of toxicity is attributed to the tumor-localized conversion of 5-FC to 5-FU (limiting systemic 5-FU exposure and the cancer-selectivity of TRAIL). This supports the notion that our therapy achieved enhanced antitumor efficacy without overt systemic side effects.

## 3. Discussion

In this proof-of-concept study, we demonstrate that genetically engineered human ADSCs delivering a combinatorial gene therapy (CD + sTRAIL) can inhibit the growth of castration-resistant prostate tumors in mice when coupled with the prodrug 5-FC. Our findings support the concept that a dual-mechanism approach—inducing cancer cell death through both 5-FU chemotherapy (via CD/5-FC conversion) and TRAIL-mediated apoptosis—yields enhanced antitumor efficacy compared to a single-mechanism (CD/5-FC alone) strategy.

We utilized an hTERT-immortalized ADSC line (ASC52-Telo) as the cellular vehicle. The hTERT immortalization circumvents the issue of primary MSC senescence, enabling the generation of a stable, clonal cell line that can be expanded to therapeutic quantities. A concern with any immortalized cell line is potential malignant transformation. We did not observe any transformation signs in vitro: our hTERT-ADSC.CD.sTRAIL cells maintained normal MSC morphology, surface marker expression. They required attachment for growth and did not form colonies in soft agar, suggesting they did not acquire anchorage-independent growth, a hallmark of tumorigenicity. This is consistent with the literature reporting that hTERT alone does not induce a tumorigenic conversion of MSCs. Telomerized adipose MSCs retained a normal karyotype and phenotype over extended culture. However, rare instances of late-onset transformation have been documented in telomerase-immortalized fibroblasts under certain conditions [23,24], underscoring the need for careful safety evaluation. In our case, we chose an ATCC-registered hTERT-MSC line and further engineered it; while we have not performed in vivo tumorigenicity assays of these cells yet, we did not observe tumor formation or ectopic tissue masses in any treated mice up to 4–6 weeks. Moving forward, we plan to conduct long-term safety studies: injecting the engineered ADSCs into immunodeficient mice without 5-FC to confirm that they do not form tumors or other lesions over several months. Additionally, we will perform karyotype or SNP-array analysis on early vs. late-passage cells to ensure genomic stability. These steps will be essential before clinical translation, given that an hTERT-immortalized cell therapy must be proven safe.

Tumor tropism and homing: The ability of MSCs to home tumors is a cornerstone of our delivery strategy. Our in vitro migration assays showed that ADSC.CD.sTRAIL cells actively migrate toward PC3 tumor-conditioned signals, similar to unmodified ADSCs. We observed the upregulation of SDF-1 and c-Kit in the engineered ADSCs, which may enhance homing since SDF-1/CXCR4 and SCF/c-Kit axes are known to mediate MSC tropism and tumor–stroma interactions [25,26,27,28,29]. In vivo, we delivered the cells via intracardiac (left ventricular) injection to disseminate them systemically and allow homing via the arterial circulation, because this route bypasses immediate lung entrapment that occurs with intravenous injection [8,9,11,21,25,26]. Although we did not directly track the ADSCs in vivo using optical imaging or cell-specific qPCR, the therapeutic outcome strongly implies that the cells reached the tumors, as significant tumor suppression was observed only in cell-injected groups. It is well-established that systemically injected MSCs can home to tumors; for instance, Hung et al. used PET imaging to show MSCs localizing to microscopic tumor foci in vivo [5]. Nevertheless, our evidence of homing is indirect. In future experiments, we will incorporate a cell-tracking modality (luciferase-expressing ADSCs for bioluminescence imaging, or labeling cells with iron nanoparticles for MRI) to directly visualize and quantify engraftment of therapeutic cells in the tumor versus other organs. We will also assess the biodistribution of cells by quantitative PCR for a human-specific gene in various organs. These studies will answer where the ADSCs go after intracardiac injection and how long they survive. It is worth noting that even if only a small fraction of injected ADSCs reach the tumor, that may suffice, as they can locally convert 5-FC to 5-FU and secrete TRAIL in situ, amplifying an apoptotic signal within the tumor microenvironment.

In our treated mice, we did not detect overt cell trapping pathology in lungs or elsewhere and no respiratory distress or organ enlargement was noted. But more sensitive detection such as histology for human vimentin-positive cells, will be performed to confirm minimal off-tumor lodging. Immune clearance of human ADSCs in nude mice which lack T cells but have NK cells is another factor. ADSCs likely persisted for some days to exert effect but may not survive long-term. This transient persistence could actually be a safety advantage.

Our approach harnesses a two-pronged mechanism. Local chemotherapy from CD/5-FC and induction of apoptosis via TRAIL. The data support that both mechanisms were at play: In vivo, the ADSC.CD + 5-FC group (without TRAIL) did show tumor growth delay compared to control, confirming that 5-FU was being produced in tumors and had an effect. However, the ADSC.CD.sTRAIL + 5-FC group showed greater tumor regression, indicating an additional contribution from TRAIL-induced apoptosis. The 5-FC dosing regimen (500 mg/kg IP daily) we used has been reported in prior studies to achieve plasma 5-FC levels in the 0.1–0.3 mM range [11,13], which, in the presence of CD-expressing cells, can yield micromolar levels of 5-FU locally.

We did not measure intratumoral 5-FU concentration in this study, which is a limitation. However, 5-FU is a diffusible drug and likely acted on neighboring tumor cells once produced. In future, we will perform HPLC or LC-MS on tumor extracts to quantify 5-FU. We acknowledge in the text that the mechanism of action is presumed: we presume that tumor-localized conversion of 5-FC to 5-FU occurred and was sufficient to kill cancer cells, and that TRAIL was secreted and induced apoptosis in a paracrine fashion. The evidence for this presumption includes the in vitro HPLC and apoptosis assays, and the known synergy between 5-FU and TRAIL reported in the literature. Li et al. showed 5-FU sensitizes gastric cancer cells to TRAIL by downregulating anti-apoptotic signals [20].

On a molecular level, 5-FU primarily triggers intrinsic apoptosis (mitochondrial pathway) by causing DNA damage and p53 activation, which upregulates pro-apoptotic BAX and downregulates BCL-2. TRAIL triggers the extrinsic pathway by binding death receptors (DR4/DR5) and activating caspase-8, which then cleaves downstream caspases (and can also cleave BID to connect to the intrinsic pathway). We saw evidence of the intrinsic pathway (increase BAX, decreased BCL-2 in tumors and co-cultures). We attempted to detect caspase-3 activation as a common downstream marker; while results were inconclusive due to technical issues, it is reasonable to assume caspases were activated. We plan to use a cleaved caspase-3 specific antibody in the future to confirm. We also did not directly assess DR4/DR5 levels or other TRAIL pathway indicators in vivo. This can be done via immunohistochemistry on tumor sections in follow-up studies. The combination of mechanisms might also help circumvent resistance: many CRPC cells, including PC3, can be somewhat resistant to TRAIL alone (due to high BCL-2 or mutations in death receptor pathways), but 5-FU can lower that threshold by upregulating DR5 or downregulating FLIP, as noted in prior studies [21,22]. By delivering both agents together, we aimed to ensure that if a tumor cell resisted one mode of killing, the other would still affect it. The net result in our study was a potent anticancer effect.

The magnitude of tumor inhibition observed with ADSC.CD.sTRAIL + 5-FC in our model was substantial. Tumors regressed in size in most treated mice. This is encouraging for a first demonstration. However, we must consider the context: subcutaneous tumor xenografts are easier to treat than diffuse metastases, and PC3 cells lack functional androgen receptor (AR), representing only one subset of CRPC (AR-independent). We have now tempered the efficacy to clarify that this is a preclinical proof of concept. We do not claim that the treatment would definitively work in all CRPC patients without further evidence. Instead, our results warrant further investigation in more clinically relevant models. Specifically, we have added text outlining plans to test the approach in: Bone metastatic CRPC models. Injecting PC3 or other prostate cancer cells into the left cardiac ventricle or tibia of mice to generate bone lesions, which more closely mimic human CRPC metastasis. We will evaluate if the ADSC therapy can home to bone and inhibit tumor growth in that microenvironment. Homing to bone might involve additional factors. We have the plan for AR-positive models using LNCaP-derived C4-2B cells which metastasize to bone and are castration-resistant but AR-positive or Enzalutamide-resistant variants of LNCaP. These models will test if our therapy is effective when androgen receptor signaling is present. TRAIL and 5-FU should theoretically work irrespective of AR status, but AR-positive tumors might have different apoptosis profiles. It will be important to show broad efficacy.

On the safety side, 5-FC is generally well-tolerated; it can cause gut microbiota changes or mild reversible bone marrow suppression at high doses, but our mice showed no illness. We did not perform blood tests in mice, which is a limitation. We clarify that no “overt toxicity” means no deaths and we acknowledge that a detailed toxicological assessment, such as histopathology of organs, and blood work was not done. Before clinical translation, such studies would be needed, of course.

Comparison to other approaches: Our strategy is related to other cell-based gene therapies. Kucerova et al. in 2007 used human adipose MSCs with a yeast CD/UPRT gene for colon cancer [13]; Kim et al. (2021) used TRAIL-expressing ADSCs with the chemotherapeutic irinotecan in a CRPC model [21]. Our contribution is in combining the prodrug/enzyme therapy with TRAIL in one platform. To our knowledge, this specific combination (CD/5-FC + TRAIL via ADSCs) has not been reported before for prostate cancer. This study has the novelty of combining these modalities. Furthermore, our use of hTERT-immortalized ADSCs as a consistent cell source is aimed to facilitate eventual clinical development, since primary MSCs vary donor-to-donor. We stress, however, that extensive safety testing and possibly insertion of a “kill switch” (such as an inducible caspase or suicide gene to eliminate the therapeutic cells if needed) might be necessary if using an immortalized cell line in humans.

We used one cell line (PC3) in nude mice with a small *n* = 5 per group. This yields preliminary data but not definitive proof of efficacy across the heterogeneous spectrum of CRPC. We mention the need to validate in additional models and to increase sample sizes for statistical power. We also did not perform a power calculation prior to the experiment. We now explicitly acknowledge that and have in fact performed a post hoc power analysis indicating that, with the variance observed, more mice per group would be needed to robustly confirm the ADSC.CD effect vs. control. Our study endpoint was 2 weeks of treatment. We do not know if tumors would regrow after that, or if any delayed toxicities could occur. We plan longer-term studies (monitoring mice for 1–2 months, performing repeat dosing cycles, etc.) to see if we can achieve complete remissions or if tumors eventually escape. We did not measure intratumoral drug levels or do detailed apoptosis pathway analysis in vivo. Our model was immunodeficient (nude mice). In an immunocompetent host, human ADSCs would be xenogeneic and likely rejected; even autologous or allogeneic human ADSCs might interact with the immune system. We note that in clinical scenarios, if using allogeneic ADSCs, immune compatibility and persistence is an issue to consider. In future, either of the autologous patient-derived MSCs could be used.

In conclusion, our study provides a proof of concept that hTERT-immortalized ADSCs can serve as effective carriers of a dual therapeutic gene payload (CD and TRAIL) to combat prostate cancer. We demonstrated efficient tumor targeting, potent cytotoxic effects on cancer cells in vitro, and significant tumor growth inhibition in vivo when combined with 5-FC prodrug administration. This combined gene therapy strategy leverages the localized activation of chemotherapy (5-FU from 5-FC) and the induction of apoptosis via TRAIL, achieving enhanced antitumor efficacy with no observed systemic toxicity in our model. The results highlight the potential of CD/sTRAIL-expressing ADSCs as an innovative cell-based treatment for castration-resistant prostate cancer.

However, we emphasize that these findings are preliminary. The therapeutic benefit was shown in a controlled small-animal setting; hence, *further studies are needed* to translate this approach. We have now bounded our conclusions to reflect that this is an early-stage investigation. We outlined the next steps, including testing in bone metastasis models and AR-positive tumors, performing more comprehensive safety profiling, and eventually moving toward clinical trial design considerations. Ultimately, this approach could pave the way for a new therapeutic avenue in advanced prostate cancer that capitalizes on the tumor-homing ability of stem cells to deliver combination gene therapy directly to tumor sites.

## 4. Materials and Methods

### 4.1. Cell Lines and Culture Conditions

Human castration-resistant prostate cancer PC3 cells (Korean Cell Line Bank, Seoul, Republic of Korea) and hTERT-immortalized human adipose-derived mesenchymal stem cells (hTERT-ADSCs; ASC52-Telo, ATCC SCRC-4000) were used. PC3 and hTERT-ADSCs were maintained in Dulbecco’s Modified Eagle Medium (DMEM; Gibco, Seoul, Republic of Korea) supplemented with 10% heat-inactivated fetal bovine serum (FBS; Gibco), 2 mM L-glutamine, 100 U/mL penicillin, and 100 µg/mL streptomycin. Cultures were incubated at 37 °C in a humidified atmosphere of 5% CO2. Cells were passaged with trypsin-EDTA upon reaching ~80–90% confluence. All cell lines were periodically tested to confirm the absence of mycoplasma contamination.

### 4.2. Generation of hTERT-ADSC.CD.sTRAIL Cells

To engineer ADSCs co-expressing CD and sTRAIL, we employed lentiviral gene transduction. A lentiviral transfer plasmid (CLV-Ubic) encoding the *E. coli* CD gene (cytosine deaminase, Gene ID: 3096544) was used to produce CD-expressing virus. Separately, a lentiviral vector encoding the human sTRAIL gene under a ubiquitous promoter was prepared. Recombinant lentiviruses were generated by transient transfection of 293T packaging cells using calcium-phosphate co-precipitation. Viral supernatants were harvested 16–20 h post-transfection and filtered to create high-titer stocks.

hTERT-ADSCs were first transduced with the CD lentivirus in growth medium containing 8 µg/mL polybrene (Sigma, Seoul, Republic of Korea) to enhance infection. After 4–6 h, the medium was replaced with fresh complete medium and cells were incubated for 48 h. Transduced cells were selected by adding 3 µg/mL puromycin (Sigma) for 7 days, yielding a stable CD-expressing ADSC line (hTERT-ADSC.CD). To introduce the sTRAIL gene, hTERT-ADSC.CD cells were subsequently infected with the sTRAIL lentivirus (also in 8 µg/mL polybrene) and selected in 5 µg/mL blasticidin (InvivoGen, San Diego, CA, USA) for 10 days. Resistant colonies were expanded to establish the dual gene-modified ADSC.CD.sTRAIL cell line. No luciferase or other reporter gene was incorporated for cell tracking in vivo, and thus no biodistribution imaging was performed in this study.

Integration and expression of the transgenes were confirmed at both the DNA and RNA levels (Table 1). Quantitative real-time PCR was used to measure transcript levels of CD and sTRAIL, using GAPDH as an internal control. Precise quantification of TRAIL in conditioned media or in vivo tumor tissues was not pursued in this study (no intratumoral TRAIL levels were measured).

### 4.3. Phenotypic Characterization of Engineered ADSCs

Mesenchymal Surface Markers: The parental and genetically modified ADSCs were analyzed by flow cytometry to verify retention of the mesenchymal stem cell phenotype. Cells were stained with antibodies against canonical MSC surface markers CD29, CD90, and CD105, as well as hematopoietic lineage markers CD34 and CD45 (all antibodies 1:50 dilution; Origene or Invitrogen, Rockville, MD, USA). Stained cells were acquired on a BD FACS Canto II flow cytometer and data were analyzed with FSC Express 7 software. hTERT-ADSC.CD.sTRAIL cells exhibited strong positivity for CD29, CD90, CD105 and were negative for CD34 and CD45, identical to unmodified hTERT-ADSCs, confirming a preserved MSC immunophenotype. The percentage of marker-positive cells was determined after subtracting isotype control signals.

Transgene and Pathway Protein Analysis: To verify pro-apoptotic pathway readiness, we probed for key apoptosis regulators. ADSC.CD.sTRAIL cells (with or without 5-FC exposure in vitro) were analyzed for BAX, BCL-2, and cleaved Caspase-3 protein levels by Western blot. Primary antibodies for BAX, BCL2, and cleaved Casp-3 (Santa Cruz, Dallas, TX, USA, 1:1000) were applied, followed by appropriate secondary antibodies. Enhanced BAX and cleaved caspase-3 with reduced BCL-2 in 5-FC-treated ADSC.CD.sTRAIL would indicate activation of the suicide gene pathway. Images were analyzed by densitometry to quantify relative protein expression. It should be noted that we did not perform flow cytometric Annexin V staining or caspase-9 activity assays to directly measure apoptosis, nor did we examine death receptor (DR4/DR5) expression on PC3 cells.

### 4.4. 5-FC to 5-FU Conversion Assay (HPLC)

The enzymatic conversion of the prodrug 5-fluorocytosine (5-FC) to 5-fluorouracil (5-FU) by the CD-expressing ADSCs was evaluated using high performance liquid chromatography (HPLC). Briefly, ADSC.CD.sTRAIL cells (and control ADSCs) were incubated with 1 mM 5-FC for 24 h, after which the culture medium was collected and processed for HPLC analysis. The solution strengths of 5-FC and 5-FU were quantified. Prior to HPLC, molecules were transformed into a lactone form by acidification. Acidified methanol, comprising 5 μL 1 N HCI/mL methanol, was used to dilute the samples. These underwent centrifugation (14,000× *g*, 2 min). The resulting supernatant was passed at a flow rate of 1 mL/min through a 4 μm Nova-Pak C18 column, of dimensions 300 × 3.9 mm, for which 75 mM ammonium acetate, 25% acetonitrile (pH 4.0) had been used for equilibration. Elution of 5-FC occurred under these parameters after 4.3 min, and 5-FU, after 6.3 min. A Jasco 82 1-FP fluorescence detector was utilized for product identification, using excitation and emission wavelengths of 375 nm and 550 nm, respectively. System Gold software (32 Karat Software, version 3.0) was employed for data analysis. Detection limits were 20 pg/pA for 5-FC and 2 pg/pA for 5-FU.

### 4.5. In Vitro Co-Culture Cytotoxicity Assays

We assessed the therapeutic efficacy of the ADSC.CD.sTRAIL/5-FC system in vitro using prostate cancer PC3 cells as targets. Cell viability assays were performed with PC3–ADSC co-cultures. PC3 cells were seeded in 96-well plates (5 × 10^3^ cells per well). After 24 h, ADSCs were added to the wells at a ratio of 20:1 (PC3:ADSC; i.e., 5% the number of PC3) to mimic a minimal effector cell presence. Co-cultures included the following conditions: (1) PC3 + unmodified ADSCs, (2) PC3 + ADSC.CD.sTRAIL, and (3) PC3 alone, each set ± 5-FC treatment. 5-FC (Sigma) was added to a final concentration of 5 µM (a dose shown to affect CD-expressing cells) and incubated for 72 h. Cell viability was then measured by a modified MTT assay (CellTiter 96, Promega, Madison, WI, USA) which detects the conversion of MTT tetrazolium to formazan by mitochondrial dehydrogenases. After treatment, 10 µL of MTT reagent (5 mg/mL) was added per well and incubated for 4 h at 37 °C. Formazan crystals were solubilized with DMSO, and absorbance was measured at 570 nm using a microplate reader. Viability for each condition was calculated as a percentage of untreated control (PC3-only) wells. Each experiment was performed in quadruplicate, and data are presented as mean ± standard error (SE).

For apoptosis assessment in co-cultures, we evaluated molecular markers of apoptosis in PC3 cells. After 72 h co-culture with or without 5-FC, PC3 cells were collected and analyzed by Western blot for pro-apoptotic BAX and cleaved Caspase-3, and anti-apoptotic BCL-2. As PC3 and ADSCs are of human origin, we did not employ species-specific antibodies; instead, the mixed cell lysates were probed, and the signal largely reflected PC3 responses given their predominance in co-culture. Co-cultures with ADSC.CD.sTRAIL + 5-FC showed increased BAX and cleaved caspase-3 and decreased BCL-2 in PC3 cells compared to controls (PC3 + 5-FC alone or PC3 + parental ADSC + 5-FC). No Annexin V staining was performed on co-cultured cells, as apoptosis was inferred from these molecular markers rather than by flow cytometric quantification of apoptotic cells, which is noted as a limitation.

### 4.6. In Vivo CRPC Xenograft Model

All animal experiments were conducted in accordance with the National Institutes of Health Guide for the Care and Use of Laboratory Animals and were approved by the Institutional Animal Care and Use Committee (IACUC) of Soonchunhyang University Seoul Hospital (Approval No. 2019-4). Male athymic nude mice (BALB/c nu/nu, 6–8 weeks old, OrientBio, Seongnam-si, Republic of Korea) were used for tumor xenograft studies. Mice were housed in a temperature-controlled environment with a 12 h light/dark cycle and given food and water ad libitum.

To establish tumors, PC3 cells (1.0 × 10^6^ in 100 µL PBS) were injected subcutaneously into the right flank of each mouse. Tumor growth was monitored by caliper measurements, and tumor volume (mm^3^) was calculated as (length × width^2^)/2. When tumors reached ~50–100 mm^3^ (approximately 2 weeks post-inoculation), mice were randomly assigned to treatment groups (5 mice per group). The treatment groups were:

1. PBS control: no therapeutic cells, no prodrug (vehicle only).
2. 5-FC alone: prodrug 5-FC administration without ADSC therapy.
3. ADSC.CD.sTRAIL alone: ADSC.CD.sTRAIL cell therapy without 5-FC (to assess the effect of TRAIL delivery alone).
4. Combination therapy: ADSC.CD.sTRAIL cell therapy plus 5-FC prodrug.

For groups receiving cell therapy, hTERT-ADSC.CD.sTRAIL cells were delivered systemically via intracardiac injection (1.0 × 10^5^ cells in 100 µL PBS, under isoflurane anesthesia). Intracardiac (left ventricular) injection was chosen to allow widespread distribution and tumor homing of the ADSCs. Control mice received an equal volume of PBS via intracardiac route. Following cell (or PBS) injection, the 5-FC prodrug was administered intraperitoneally at 500 µg/kg/day. A cyclical dosing regimen was used: 5 consecutive days of 5-FC injections followed by a 2-day break, then another 5-day treatment course (for a total of 10 doses over 12 days). Mice in the 5-FC alone and combination groups received 5-FC, whereas control and ADSC-only groups received sham IP injections of PBS on the same schedule. Tumor sizes were measured at the start of treatment (day 0) and then twice weekly. After 14 days from the start of therapy, mice were humanely euthanized by CO_2 asphyxiation. Tumors were harvested, measured, and weighed. No bioluminescence or fluorescent tracking of the administered ADSCs was performed in vivo (i.e., ADSCs were not luciferase-labeled), so the biodistribution and survival of injected cells were not directly monitored, which is a limitation of the present study. Additionally, intratumoral levels of 5-FU or TRAIL were not quantified; therapeutic efficacy was inferred from tumor response endpoints without measuring drug concentrations in tissues.

### 4.7. Statistical Analysis

Quantitative data are presented as mean ± standard error (SE) unless stated otherwise. In vitro viability comparisons between groups were analyzed by a two-tailed Mann–Whitney U test (non-parametric). For the in vivo tumor growth data, two-way analysis of variance (ANOVA) with repeated measures was used to compare tumor volume trajectories among the groups, followed by Tukey’s post hoc test for multiple comparisons. Final tumor volumes between groups were additionally compared by one-way ANOVA. A *p* < 0.05 was considered statistically significant. All statistical analyses were performed using SPSS 25.0 (IBM Corp., Armonk, NY, USA) or GraphPad Prism 9. Data visualization was done with GraphPad Prism.

## 5. Conclusions

Human adipose-derived stem cells engineered to co-express CD and sTRAIL demonstrated efficient tumor-tropic migration, potent cytotoxic effects on prostate cancer cells in vitro, and significant tumor growth inhibition in vivo when combined with the 5-FC prodrug. This combined gene therapy strategy leverages the bystander effect of 5-FU and the apoptotic action of TRAIL, achieving enhanced antitumor efficacy with minimal systemic toxicity while allowing control of the therapeutic cells. The results of this study highlight the potential clinical utility of CD/sTRAIL-expressing ADSCs as an innovative treatment for castration-resistant prostate cancer.

## Figures and Tables

**Table 1:** Sequence of PCR primers.

Gene	Sequence	Size (bp)
*CD*	Sense: 5′-GCGCGAGTCACCGCCAGCCACACCACGGC-3′ Antisense: 5′-GTTTGTATTCGATGGCTTCTGGCTGC-3′	559
*sTRAIL*	Sense: 5′-CAACTCCGTCAGCTCGTTAGAAAG-3′ Antisense: 5′-CGGCCCAGAGCCTTTTCATTC-3′	200
*SCF*	Sense: 5′-TCATTCAAGAGCCCAGAACC-3′ Antisense: 5′-CTGCCCAGTGTAGGCTGGA-3′	190
*c-kit*	Sense: 5′-GTGAATGGCATGCTCCAATG-3 Antisense: 5′-GTGCCATTGTGCTTGAATGC-3′	200
*SDF-1*	Sense: 5′-CCTTGTGAGAGATGAAAGGG-3′ Antisense: 5′-AAATGCAGGGTCTAAATGCTG-3′	130
*CXCR4*	Sense: 5′-AAATCTTCCTGCCCACCATCT-3′ Antisense: 5′-GCCTTGCATAGGAAGTTCCC-3′	220
*VEGF*	Sense: 5′-TCATGGATGTCTATCAGCGC-3′ Antisense: 5′-TGATGTTGGACTCCTCAGTG-3′	180
*VEGFR1*	Sense: 5′-GGAACAAGGCAAGAAACCAA-3′ Antisense: 5′-TGGAAGACAGGAACTCCATG-3′	200
*VEGFR2*	Sense: 5′-ATCCCTGTGGATCTGAAACG-3′ Antisense: 5′-GATGCCAAGAACTCCATGCC-3′	200
*VEGFR3*	Sense: 5′-CCCGACCTTGAACATCACGG-3′ Antisense: 5′-ACACCTTGCAGTAGGGCCTG-3′	200
*GAPDH*	Sense: 5′-TGCACCACCAACTGCTTAGC-3′ Antisense: 5′-CCATCACGCCACAGTTTCC-3′	116

## Data Availability

The data presented in this study are available on reasonable request from the corresponding author (Y.S.S.).
